# Neuroprotection by rAAV-mediated gene transfer of bone morphogenic protein 7

**DOI:** 10.1186/1471-2202-15-38

**Published:** 2014-03-11

**Authors:** Ann-Marie Heinonen, Mahbubur Rahman, Godwin Dogbevia, Hannah Jakobi, Stefan Wölfl, Rolf Sprengel, Markus Schwaninger

**Affiliations:** 1 Max Planck Institute for Medical Research, Jahnstrasse 29, Heidelberg D-69120, Germany; 2 Institute of Experimental and Clinical Pharmacology and Toxicology University of Lübeck, Lübeck, Germany; 3 Institute of Pharmacy and Molecular Biotechnology, Department of Biology Heidelberg University, Heidelberg, Germany

**Keywords:** Stroke, Apoptosis, BMP7, BMP2, Noggin, 2A peptide, Ribosomal skip

## Abstract

**Background:**

Bone morphogenic proteins (BMPs) promote the survival of neurons, suggesting a therapeutic application of BMPs in the treatment of acute and chronic neurodegenerative disorders. However, the application of recombinant BMPs *in vivo* is limited by their short half-life. To provide a continuous supply for functionally active BMPs, we expressed BMP7, BMP2 and the BMP inhibitor Noggin under the control of rAAV vectors *in vivo.* For visual control of rAAV-mediated BMP (v-BMP) expression we fused the secreted morphogenic polypeptides and the fluorescent reporter protein Venus via the ‘ribosomal skip’ promoting 2A peptide-bridge.

**Results:**

In primary cortical neurons, the rAAV-expressed morphogenic polypeptides were efficiently released from the 2A-Venus fusion precursors, were secreted, correctly processed and functionally active as shown by their effects on Smad phosphorylation in HeLa cells and in primary neurons, by the protection of v-BMP7-transduced primary cortical neurons against oxidative stress, and by the activation of BMP responsive GFP in v-BMP2 transduced reporter mice. In the stroke model of middle cerebral artery occlusion rAAV-transduced v-BMP7 reduced the infarct size in mice.

**Conclusion:**

Polycistronic rAAV vectors encoding secreted polypeptides and 2A-linked reporter proteins are potential novel therapeutic tools for the treatment of neurological and neurodegenerative diseases. Using this technique we documented that rAAV delivery of BMP7 reduced ischemic cell death in mice.

## Background

Bone morphogenic proteins (BMP) are members of the transforming growth factor-β (TGFβ) family. Mammalians encode and express more than a dozen BMPs. Two members, BMP2 and BMP7, are in clinical use for the treatment of bone fractures providing evidence for the therapeutic application of BMPs to regulate the viability and differentiation of osteoblasts [1], which might be of strong interest for other human cell types; in particular for those in the nervous system. BMPs play an important role during development of the nervous system [2]. In adults, they stimulate adult neurogenesis, exert a neuroprotective effect, and stimulate the regeneration of neurons [3-5]. In models of ischemic stroke BMP7 reduced the infarct size [4,6,7] and improved functional recovery at delayed time points after stroke [3,4,7-9]. BMP7 also exerted neuroprotective effects in models of Parkinson’s disease, spinal cord injury [10,11]. In addition, BMP2 had neuroprotective properties *in vitro*[12,13] and stimulated angiogenesis, neuronal differentiation, and neurite outgrowth [14-16]. Although BMP2 is upregulated after cerebral ischemia [17], its function as an endogenous neuroprotectant is controversial since recombinant soluble Noggin, an antagonist of BMP2, BMP4 and BMP7, demonstrates neuroprotective effects in models of stroke [18-20].

A major obstacle for the therapeutic use of BMPs is the short half-life of the peptides; in the case of BMP7 about 30 min [21], precluding intravenous administration for chronic conditions. Transplanted cells secreting BMPs or Noggin can be one option to solve this issue [22,23] but viral gene therapy using lentiviruses or recombinant adeno-associated viruses (AAVs) might open an attractive, well-defined alternative option for providing constant supply of recombinant BMP [24,25].

In this study we used rAAVs to express BMP2, BMP7, and Noggin in the brain of mice since rAAVs enable efficient gene delivery in the CNS without inducing toxic or inflammatory responses [26]. For visual inspection of rAAV encoded gene transduction we expressed BMP2, BMP7, and Noggin as fusion with a fluorescent protein (FP) using the ribosomal skipping 2A peptide bridge from *Thosea asigna* virus [27]. In contrast to conventional techniques, such as the internal ribosomal entry site (IRES), bidirectional and dual promoters, or co-infection of several viruses, the ribosomal skipping leads to nearly equimolar ratios of the 2A-linked proteins [28-31].

In this study we demonstrate that BMP2, BMP7, or the inhibitor Noggin can be functionally expressed by rAAV encoded, 2A fused open reading frames together with fluorescent reporter proteins. When released from the 2A linked reporter proteins during protein translation, BMP2, BMP7, and Noggin are secreted and are functionally active. We found that rAAV-delivered BMP7 exerted neuroprotective properties *in vitro* and *in vivo* suggesting that this might represent a new approach for long-term administration of BMP7 in neurological diseases.

## Results

### Expression of secreted morphogenic proteins and 2A-linked Venus

**Figure 1:**
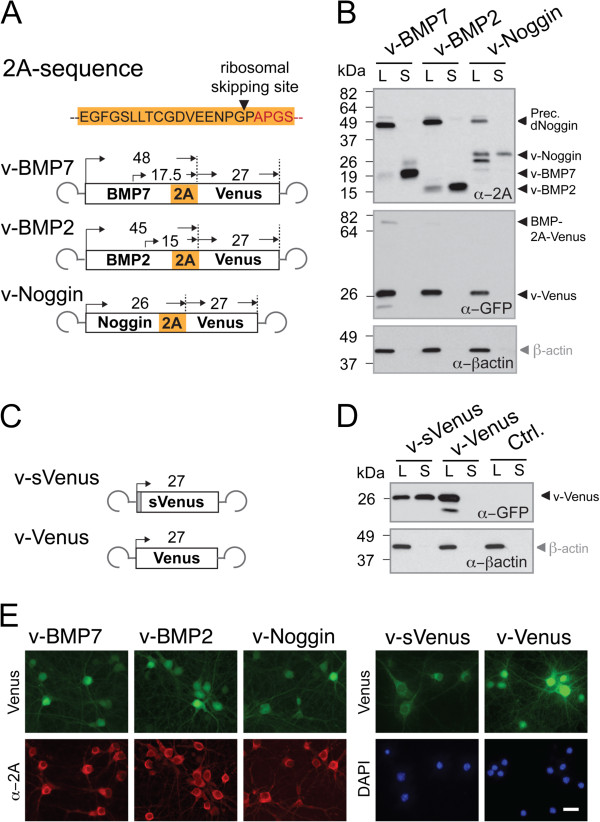
**Coexpression and release of secretory proteins and Venus via the 2A peptide bridge in cultured neurons. A**. Top, amino acid residues of the 2A peptide from *Thosea asigna* virus (T2A). The ribosomal skipping site (lack of peptide bridge formation between G and P) is indicated by a black arrowhead. Four amino acids (red) were added to create a constant C-terminal environment among different constructs. Bottom, schematic diagram of rAAVs. The theoretical molecular weights for the expected proteins of the rAAV encoded polypeptides are indicated in kDa above the boxed open reading frames. **B**. Primary cortical neurons (PCN) were infected with rAAVs expressing v-BMP7, v-BMP2 and v-Noggin. Immunoblots revealed BMP2 and BMP7 precursors and mature secretory morphogenic proteins and some BMP7-2A-Venus and BMP2-2A-Venus full-length proteins in lysates (L). In PCN supernatants (S) only mature morphogenic proteins could be detected. Venus was visible in PCN lysates and not in the corresponding supernatants. Venus was detected by anti-GFP antibodies. Anti-beta-actin was used as loading control. **C**. Schematic drawing of rAAVs encoding secreted Venus (sVenus) and cytosolic Venus. **D**. Immunoblots of PCN expressing rAAV-Venus (v-Venus) and rAAV-sVenus (v-sVenus). The v-Venus was not present in PCN supernatants, whereas sVenus was observed in lysates and supernatants using anti-GFP antibodies. **E**. After infecting PCNs with the indicated rAAV constructs immunocytology using anti-2A and anti-GFP antibodies showed co-expression of cytosolic Venus (green) and nuclear excluded BMPs or Noggin (red). On the right the expression of v-sVenus and v-Venus is depicted. Cell nuclei are stained with DAPI (blue) indicating the high efficiency of rAAV infection. Scale bar, 20 μm.

To analyze whether BMP2, BMP7 and Noggin can be functionally expressed in rAAV encoded gene transfer experiments we generated several rAAV vectors by linking the secretory proteins BMP7, BMP2 and Noggin via the 2A peptide to the cytosolic fluorescent protein Venus [32] or to tdTomato (not shown) [33] in a single open reading frame (Figure 1A).

For the functional analysis of the rAAV vectors in neurons we first infected primary neurons by rAAVs encoding the viral BMPs (v-BMP2 and v-BMP7) and viral Noggin (v-Noggin) (Figure 1A). Seven days post infection (DPI 7) cell culture medium (supernatant, S) contained a major amount of mature v-BMP7, v-BMP2, and v-Noggin as revealed by immunoblots using an anti-2A antibody selective for 18 amino acids of 2A-peptides that remained at the C-terminus of v-BMP2, v-BMP7 and v-Noggin after successful ribosomal skipping (Figure 1B). For Noggin, three bands were visualized in immunoblots of cell lysates corresponding to the unmodified protein (26 kDa), the glycosylated protein (30 kDa), and the mature protein (32 kDa). In addition, in cell lysates the anti-2A antibody detected the BMP7 and BMP2 precursors, in the range of the predicted molecular weight (MW) of 48 kDa and 45 kDa, respectively. The anti-2A antibody also detected a Noggin-immunosignal at about 53 kDa, which might indicate residual Noggin-2A-Venus fusion proteins that escaped the ribosomal skip. However, the anti-GFP antibody revealed only minor amounts (about 6%) of BMP7-2A-Venus and BMP2-2A-Venus full-length proteins but no Noggin-2A-Venus fusion. This suggests that the 53-kDa immunosignal for Noggin in the supernatant represents Noggin dimers that can be detected under low stringent reducing conditions during electrophoresis [34]. Thus, the ribosomal skipping and the secretion of the target proteins encoded by the rAAVs were very efficient. The minor amount of mature factors that can be detected in the lysates of neurons is most likely due to the crude, one-step separation procedure of supernatants and lysates. In contrast to the almost complete secretion of BMPs and Noggin, the secreted Venus (sVenus) which contains the N-terminal signal sequence of Noggin (Figure 1C) was only partially secreted and equal amounts of sVenus were found in the supernatant and cell lysate using the Venus cross reactive anti-GFP antibody (Figure 1D).

The immunocytology of rAAV-infected primary cortical neurons on DPI 7 was in accordance with the concept that 2A-tagged v-BMP7, v-BMP2 and v-Noggin entered the secretory pathway in rAAV transduced cells, since the 2A immunoreactivity for all three factors showed nuclear exclusion (Figure 1E). The immunofluorescence was associated with cell membranes, whereas the signal in v-Venus transduced neurons was localized mainly in the cytosol indicating that low expression of the virally transduced factors is not the reason for nuclear exclusion of their 2A immunoreactivity. A similar change in the distribution of the Venus fluorescence was found when the fluorescence patterns of virally delivered sVenus and Venus (v-sVenus; v-Venus) were compared (Figure 1E). Therefore, we conclude that the subcellular immunosignal distributions of 2A-linked secreted and cytosolic proteins indicate an efficient secretion of the secreted proteins in 2A based polycistrons.

### Efficient expression of 2A-linked secretory proteins *in vivo*

**Figure 2:**
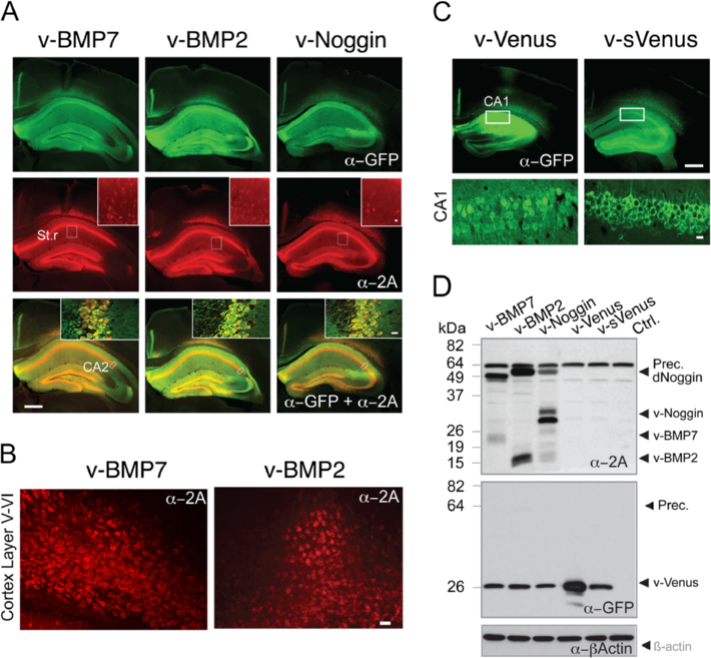
**Efficient co-expression of secretory BMPs, Noggin, and Venus in the cortex and the hippocampus of rAAV injected mice at DPI 14. A**. Confocal fluorescent immune histological images of sections from rAAV infected adult mouse brains (hippocampus and cortex) demonstrated cytosolic expression of Venus (green) and v-BMPs or v-Noggin (red) visualized by anti-GFP and anti-2A antibodies, respectively. The insets in the middle row show the expression of v-BMPs or v-Noggin in glia and neurons in the stratum radiatum (St.r) at high magnifications. In the insets of the bottom row, overlay of the CA2 region in the hippocampus is depicted demonstrating the nuclear exclusion of the 2A-tagged peptide factors (yellow) in cells expressing Venus (green) in the nucleus and the soma. A. Scale bar, 500 μm. Scale bar inserts, 15 μm. **B**. Fluorescent immune-histological images show the nuclear excluded 2A-tagged v-BMP7 and v-BMP2 in rAAV infected neurons of cortical layer V/VI. Scale bar, 20 μm. **C**. Confocal fluorescent immune-histological images of sections from rAAV infected adult mouse brains show expression of v-Venus and membrane associated v-sVenus in the hippocampus and cortex. Lower panel: High magnifications of the hippocampal CA1 region of the somatic and nuclear excluded Venus immunoreactivity. Scale bar, 500 μm. Scale bar insets, 15 μm. **D**. After rAAV infection immunoblots detected precursors and mature protein factors in hippocampal extracts. Precursors (Prec.), dimerized Noggin (dNoggin) and mature proteins are indicated by arrowheads. Beta-actin was used as loading control.

We aimed to use the rAAVs for the delivery of BMP2, BMP7 and Noggin in the adult brain. Therefore, we examined the efficiency of the 2A-mediated co-expression and ribosomal skipping of 2A-linked rAAV-encoded proteins in the brain *in vivo*. We injected rAAV-BMP7-2A-Venus, rAAV-BMP2-2A-Venus, rAAV-Noggin-2A-Venus, rAAV-Venus and rAAV-sVenus into the cortex (CTX) and hippocampus (HPC) of adult C57BL6/N mice. At DPI 14 we detected similar Venus fluorescence intensity specifically in rAAV injected brain regions for all rAAVs (Figure 2A). The immune-histological staining with anti-GFP and anti-2A antibodies showed cytosolic Venus expression whereas v-BMP2, v-BMP7 and v-Noggin immunosignals were nuclear excluded and were associated with the cell membranes (Figure 2A, B). A similar cellular distribution could be observed for v-sVenus and v-Venus in the HPC (Figure 2C). This strongly suggests that the secretion of the rAAV expressed v-BMP7, v-BMP2 and v-Noggin is indicated by their membrane associations. The cellular resolution of v-BMP2, v-BMP7 and v-Noggin in the stratum radiatum revealed that glia as well as neurons express the rAAV delivered morphogenic factors (Figure 2A). Since the ubiquitous CAG promoter controls the expression of the rAAV delivered 2A-linked morphogenic factors the astroglial expression was expected.

In crude hippocampal extracts mature v-BMP2, v-BMP7 and v-Noggin could be detected together with their v-BMP2 and v-BMP7 precursors and Noggin dimer of v-Nogin (Figure 2C). As already noticed in virally transduced primary neurons (Figure 1B), the electrophoretic mobility of the v-BMP2 precursor (at 49 kDa) is slower than expected (45 kDa). Since this slower mobility of the v-BMP2 45-kDa precursor was consistently observed in different expression systems this might be an intrinsic property of the v-BMP2 precursor in our electrophoretic system.

### 2A-released secretory proteins are biologically active

To analyze whether 2A-tagged v-BMP2, v-BMP7 and v-Noggin expressed in brain cells are bioactive we analyzed v-BMP2, v-BMP7 and v-Noggin induced Smad1/5/8 phosphorylation, the neuroprotection afforded by v-BMP7 against H_2_O_2_ toxicity and the v-BMP2 activation of the BMP2 BRE:GFP reporter gene in the BMP reporter mouse brains [35].

**Figure 3:**
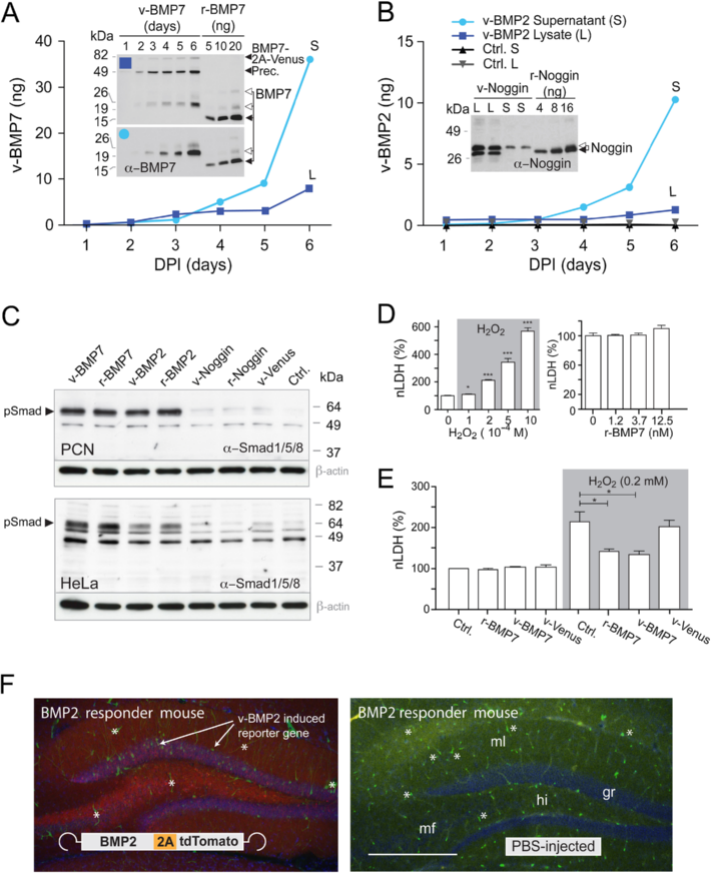
**2A-released v-BMPs and v-Noggin are biologically active. A**. BMP7 in lysates (L) and culture medium (S) of rAAV infected PCNs at different time points. Inset: Anti-BMP7 immunoblots of L (4 μl) and S (12 μl) used for quantification. **B**. Time course of v-BMP2 expression in PCN determined by ELISA of 4 μl L and 12 μl S. Inset: Noggin immunoblot of L and S of rAAV-Noggin-2A-Venus infected PCN at DPI 6. Precursor and released peptides are indicated by arrowheads; cystines containing forms by open arrows. **C**. Anti pSmad immunoblots of PCN (top) and HeLa cells (bottom) treated for 4 h with recombinant proteins or conditioned medium from rAAV infected PCNs as indicated. Ctrl.: non-infected PCN. Loading control: β-actin. **D**. left: H_2_O_2_ induced death of PCN as shown by lactate dehydrogenase (LDH) release 24 h after adding H_2_O_2_. Values are means ± SEM, One-way ANOVA, p < 0.0001. *p < 0.05, ***p < 0.0001 in comparison to untreated groups. **D**, **E**. Pretreatment with r-BMP7 (1.25 nM) and rAAV-mediated v-BMP7 expression reduced H_2_O_2_ induced LDH release in PCN. R-BMP7 was added 30 min before H_2_O_2_. One-way ANOVA, p < 0.0006. *p < 0.05 (Tukey’s posthoc test). LDH is given relative to controls (Ctrl.). **F**, V-BMP2 specific induction of the BMP responder GFP in BRE:GFP mice. RGB overlay fluorescent images of hippocampal DG region in coronal brain sections of (left) BRE:GFP mice injected with rAAV-BMP2-2A-tdTomato and (right) with PBS as control. GFP was visualized by anti-GFP (green), and cells nuclei with DAPI (blue). tdTomato auto-fluorescence is in red. Hilus region (hi); molecular layer (ml) of the dentate gyrus (DG); mossy fiber projections (mf). Arrows indicate GFP expressing cells in the granular cell layer (gr). Blood vessels show an unspecific fluorescence in both brain sections (stars). Scale bar; 0.5 mm.

First, we collected at different time point cell culture supernatants from PCN that express v-BMP2, v-BMP7, and v-Noggin and compared their immunoreactivity to commercially available morphogenic proteins. Thus, anti-BMP7 detected r- and v-BMP7 and anti-Noggin r- and v-Noggin in immunoblots (Insets, Figure 3A, B) whereas the anti-BMP2 could only be used in ELISA for v-BMP2 detection (histogram, Figure 3B) demonstrating immune-cross-reactivity of homologous recombinant and virally expressed morphogenic proteins. Most of r-BMP7 and r-Noggin were found in the fully reduced form with the expected molecular weights of 13.5 and 26 kDa, respectively. The cystines containing forms of BMP2 and Noggin exhibit reduced SDS-PAGE electrophoretic mobility and are the dominant isoforms for v-BMP2 and v-Noggin (Insets, Figure 3A, B). Substantial amounts of v-BMP2, v-BMP7 and v-Noggin B could be detected in PCN culture media when compared to the recombinant morphogenic proteins in the same immunoblot (Insets, Figure 3A, B).

We treated PCN with conditioned supernatants of PCN cultures (Figure 3A, B) since it was documented that BMP2 and BMP7 stimulate the phosphorylation of Smad1/5/8 (pSmad1/5/8) whereas Noggin inhibits the formation of pSmad [36]. As controls we applied recombinant r-BMP7, r-BMP2, r-Noggin (100 ng/ml) and supernatant from non-transfected PCN. The Smad1/5/8 specific immunoblots showed that r-BMPs and v-BMPs increased the level of pSmad 4 h after stimulation whereas supernatants from v-Noggin or v-Venus expressing cells and uninfected cells did not increase pSmad1/5/8 levels (Figure 3C; upper panel). The identical treatment of HeLa cells provided a similar result (Figure 3C; lower panel). In HeLa cells the v- and r-BMP7 stimulation induced higher pSmad1/5/8 levels than v- and r-BMP2. The v- and r-Noggin treated HeLa cells showed lower pSmad1/5/8 levels than HeLa cells stimulated with conditioned supernatants harvested from Venus expressing or uninfected PCN (Figure 3C; lower panel). Thus, the efficient stimulation of pSmad1/5/8 by v-BMP2 and v-BMP7 strongly suggested that the 2A-tagged v-BMP2 and v-BMP7 are secreted as bioactive proteins from rAAV infected PCN.

Since our goal was the application of the virally delivered BMPs for neuroprotection and since reactive oxygen species are known to be involved in the pathophysiology of several brain disorders, including stroke, we exposed primary cortical neurons to H_2_O_2_ investigating the protective activity of rAAV-delivered BMP7. On day *in vitro* (DIV) 11 we treated PCN with H_2_O_2_ and 24 h later we measured the cell toxicity indicator lactate dehydrogenase (LDH) in the supernatant of PCN cultures. As shown in Figure 3D H_2_O_2_ exposure induced neuronal toxicity in a H_2_O_2_ concentration-dependent manner. For the subsequent experiments we used 0.2 mM H_2_O_2_ that induced about 30 % of the global toxicity obtained by 1 M H_2_O_2_ exposure. Treatment with r-BMP7 itself (1.2 nM to 12.5 nM) did not affect LDH release (Figure 3D, right panel). However, treatment of DIV 11 neurons with r-BMP7 (1.2 nM) 30 min before H_2_O_2_ exposure significantly reduced H_2_O_2_-induced LDH levels in the medium (Figure 3E). Interestingly, infection of PCN with rAAV-BMP7-2A-Venus at DIV 4 also protected PCN from H_2_O_2_-induced exposure at DIV 11 (Figure 3E). These results indicate that, given shortly before the noxious stimulus or chronically administered by rAAV delivery several days before H_2_O_2_ treatment, v-BMP7 has protective properties in primary neurons.

To provide additional evidence for the bioactive expression of v-BMPs in the adult brain we injected rAAV-BMP2-2A-tdTomato into the hippocampus and cortex of BRE:GFP reporter mice [35]. Two weeks after rAAV delivery we detected the majority of GFP positive cells in the granular cell layer of the dentate gyrus (DG) in addition to some sparse GFP immunopositive neurons and glia in the cortex at the injection site (Figure 3F). The presence of the GFP immunopositive cells could not be observed in PBS-infected BRE:GFP mice (Figure 3F) suggesting that the virally delivered v-BMP2 is functionally active. The poor GFP expression by rAAV transduced v-BMP2 might reflect the low sensitivity of the BRE:GFP reporter in brain tissue, since injection of r-BMP2 did lead to GFP positive cells 24 or 48 h after injection of rBMP2 (not shown). Thus, in summary, our results collectively show that BMP2, BMP7 and can be expressed and secreted as native peptides in neuronal cultures and in adult brains as rAAV encoded 2A fusions.

### Virally expressed BMP7 has a neuroprotective effect in MCAO

**Figure 4:**
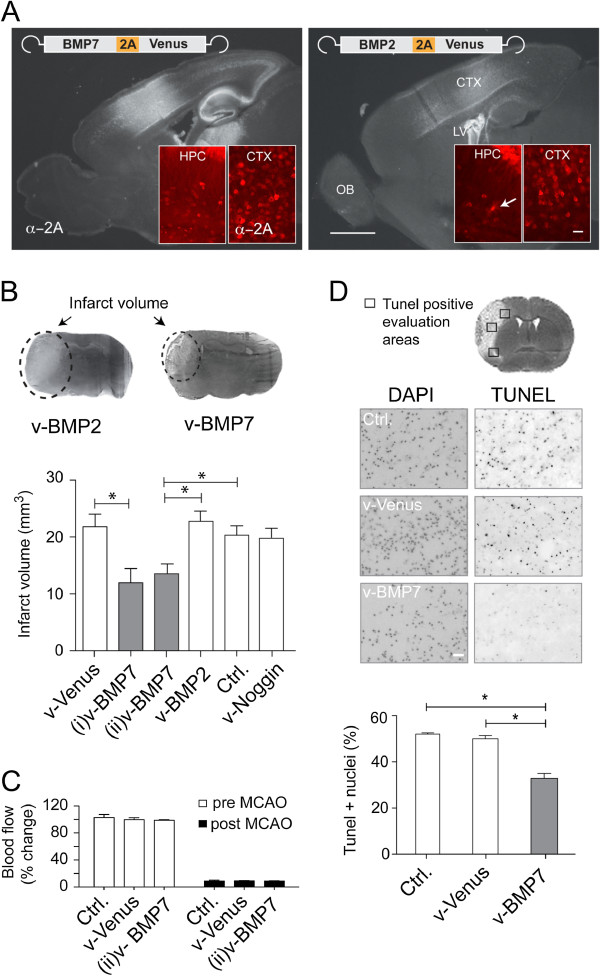
**rAAV-transduced v-BMP7 decreases ischemic brain damage. A**. Efficient, long-lasting v-BMP2 and v-BMP7 expression eight weeks after rAAV injection into the brains of neonatal mice. Fluorescent immunohistological images of sagittal brain sections are in gray scale. CTX, cortex; HPC, hippocampus; OB, olfactory bulb; LV, epithelium of lateral ventricles. Scale bar, 1 mm. Insets: confocal images of cortical neurons expressing v-BMP2 and v-BMP7. Cellular resolutions show neurons expressing v-BMP2 and v-BMP7 and one v-BMP2 positive astrocyte. Scale bar, 30 μm. **B**. Top: silver-stained coronal brain sections with marked, unstained infarcts 48 h after MCAO. Bottom: quantification of infarct volumes. The brain edema did not differ between groups. Values are means ± SEM. First experiment (i): v-Venus, n = 8; v-BMP7, n = 9; v-BMP2, n = 12; v-Noggin, n = 7); one-way ANOVA, P = 0.0049, *, p < 0.05 (Tukey’s posthoc test). Second experiment (ii): Ctrl., n = 4; v-Venus, n = 8; v-BMP7, n = 8; one-way ANOVA, P = 0.0265, *, p < 0.05 (Tukey’s posthoc test). **C**. Virally delivered v-BMP7 had no influence on cerebral blood flow measured by Doppler sonography before and after MCAO in (ii). Values were normalized to Ctrl. before MCAO; means ± SEM. **D**. The rAAV-expressed v-BMP7 reduced apoptosis after MCAO. Top: apoptotic nuclei were quantified 48 h after MCAO on TUNEL-stained coronal sections in indicated areas. Middle: images of TUNEL- and DAPI-stained (total number of nuclei) brain sections after MCAO in gray-scale. Scale bar, 50 μm. Bottom: quantification of apoptotic nuclei after MCAO was determined in relation to the total number of DAPI stained nuclei in all three indicated areas. Values are means ± SEM (n = 3). One-way ANOVA, P = 0.0002, *, p < 0.05 (Tukey’s posthoc test).

To study the neuroprotective effects of v-BMP2 and v-BMP7 in adult mice we aimed to analyze whether v-BMP2 and v-BMP7 can decrease the infarct size after occlusion of the middle cerebral artery (MCAO) in adult mice. Since the stereotactic delivery of rAAV in adult mice is restricted to the injection site of the virus (about 1 mm^3^) we injected rAAV into the lateral ventricle and the hippocampi of newborn (P0) mice for robust virally mediated gene expression [37]. Injected mice did not show behavioral abnormalities during adolescence and adulthood. In sagittal, immunhistological brain sections of P0 injected mice robust and widespread expression of nuclear excluded, membrane-attached v-BMP2 and v-BMP7 could be visualized 8 weeks after rAAV injection with strongest expression in neurons of the cortex and the hippocampus (Figure 4A). In contrast to adult rAAV injected mice (Figure 2A) the expression of the rAAV-encoded 2A-linked morphogenic factors was restricted to neurons with very few rAAV expressing astroglia (Figure 4A insets).

In a second cohort of eight-week-old mice injected at P0 with rAAV-BMP7-2A-Venus, rAAV-BMP2-2A-Venus, rAAV-Noggin-2A-Venus and rAAV-Venus MCAO was performed. In this model of ischemic strokes, infarcts are mainly limited to the cortex. When cortical infarct volumes were quantified 48 h after MCAO, rAAV-mediated overexpression of v-BMP7 (Figure 4B, (i)v-BMP7), significantly reduced infarct volumes, whereas the rAAV mediated expression of v-Venus, v-BMP2 and v-Noggin did not alter infarct volumes (Figure 4B). In another independent experimental group, we confirmed the protective effect of v-BMP7 in MCAO (Figure 4B, (ii)v-BMP7) and showed in these mice that v-BMP7 did not affect the cerebral blood flow (Figure 4C). To further investigate the protective properties of v-BMP7 *in vivo*, we analyzed neuronal apoptosis after MCAO in eight-week-old P0 rAAV-BMP7-2A-Venus injected animals. Apoptosis was evaluated in the periphery of the ischemic area by TUNEL staining on coronal sections at the level of the anterior commissure. We detected significantly fewer TUNEL-positive cells in mice expressing v-BMP7 compared to rAAV-Venus infected or naïve mice (Figure 4D).

Our results indicate that rAAV-induced neuronal overexpression of BMP7 is protective in MCAO and that rAAV-mediated polycistronic gene transfer can be applied to study cerebral ischemia *in vivo.*

## Discussion

The morphogenic protein BMP7 has neuroprotective properties *in vitro*. However, *in vivo* administration is limited by the short half-life and the large amounts of biologically active BMP7 that need to be administered (at least 10 mg/kg body weight) [8], which might explain why recombinant BMP7 reduced the infarct volume in some, but not all experimental stroke studies [4,7-9]. Using rAAV expressed BMP7 we observed a 2-fold reduction in the infarct size, suggesting that rAAV vectors represent a superior mode for the delivery of biological active BMP7. Previous work has revealed an anti-apoptotic effect of BMP7 in neurons *in vitro*[38,39]. Our work extends this finding, demonstrating that BMP7 reduces apoptotic cell death *in vivo* as rAAV expression of BMP7 significantly decreased the number of TUNEL-positive cells in the periphery of the ischemia.

In contrast to a previous study [18], the viral expression of Noggin, an extracellular inhibitor of BMP2, BMP4 and BMP7, had no effect on the infarct size although we verified that the rAAV expressed Noggin was bioactive by decreasing the level of Smad phosphorpylation of HeLa cells. Besides the different time points for the analysis after MCAO (2 days in this study versus 14 days in [18]) a lower expression level of biological active Noggin in our system cannot be excluded. Similarly, low expression levels of v-BMP2 might be responsible for the lack of neuroprotection in our MCAO experiments.

Our study was not designed to analyze long-term effects of BMP2, BMP7 and Noggin, but rather was designed to propose a convenient, reliable route for prolonged delivery of short-lived peptide hormones. Future work will be required to explore the potential of BMPs and BMP inhibitors in regenerative treatment of stroke and other neurodegenerative diseases. Similarly, a paracrine or autocrine neuroprotective activity of the v-BMP7 needs to be investigated in future studies, using neuron specific rAAV vectors.

Our study illustrates that 2A-tagged BMP2, BMP7 and Noggin can be co-expressed in 2A-linked fusions with a reporter protein in neurons using rAAVs as gene transfer vectors. In cells of adult mouse brains the 2A induced ribosomal skip [29] was very efficient and no full-length 2A-fusion protein composed of the secretory polypeptides, the 2A peptide linker and Venus could be detected in adult mouse brains. After or during translation the N-terminal secretory protein precursors and the C-terminal reporter protein were differentially sorted as separated proteins into the cytosol or functionally assembled in secretory pathways indicating that the C-terminally attached 2A peptide did not inhibit the BMP2, BMP7 and Noggin bioactivity and that v-BMP7 is similar active as r-BMP7 *in vitro*[40].

## Conclusion

Polycistronic expression of 2A-linked peptide hormones and a reporter gene using rAAVs provide a convenient and promising gene therapeutic tool for the genetic delivery of neuroprotective factors.

## Methods

### Animal experimentation

Molecular, biochemical experiments and genetic manipulations of mice were licensed by the regional council in Karlsruhe, Germany, under project licenses 35–9185.81/G171-10. Animal experiments and were performed according to the animal welfare guidelines of the Max Planck Society. Efforts were made to minimize numbers of animals used.

### Cloning strategies of specific rAAV vectors

The plasmid pAAV-CAG-Noggin-2A-Venus was generated by PCR-isolating the CMV enhancer chicken β-actin (CAG) promoter from plasmid pAAV-CMV-Rluc2A-Venus, GenbankID: KC152484 [41], with KpnI and EcoRI restriction sites and subcloning it between XbaI and EcoRI in pAAV-Syn-Noggin-2A-Venus [27] by 5′ blunt and EcoRI ligation to replace the synapsin promoter. The human BMP2 and human BMP7 genes were amplified by PCR from the pcDNA3.1-BMP2 and pcDNA5-BMP7 template DNA using specific primers to replace Noggin in pAAV-CAG-Noggin-2A-Venus using EcoRI and XhoI restriction sites to construct pAAV-CAG-BMP2-2A-Venus and pAAV-CAG-BMP7-2A-Venus. The plasmids pAAV-CAG-BMP7-2A-Tomato, pAAV-CAG-BMP2-2A-Tomato and pAAV-CAG-Noggin-2A-Tomato were generated by replacing Venus by tdTomato from pAAV-Syn-iCre-2A-Tomato using BamHI and BsrGI restriction sites. To generate pAAV-CAG-Venus, Noggin-2A was deleted in pAAV-CAG-Noggin-2A-Venus by EcoRI and BamHI restriction digest and subsequent religation of the vector.

### Generation of rAAV vectors

Recombinant AAV gene transfer vectors were generated by molecular cloning of specific gene cassettes into rAAV2-based expression vectors [42], containing viral cis-acting elements required for genome replication and packaging, a woodchuck posttranscriptional regulatory element (WPRE), a bovine growth hormone polyadenylation site (bGHpA), two flanking inverted terminal repeats (ITRs) of serotype 2 (S2) and the cytomegalovirus enhanced chicken beta-actin promoter (CAG) for efficient expression in cells of the brain. All constructs were confirmed by sequencing.

### Preparation and delivery of rAAV vectors

Recombinant AAVs of serotype 1 and 2 were generated as described [27] and purified by AVB Sepharose affinity chromatography [43]. For each virus the genomic titer was determined by real-time PCR using primers and a probe against WPRE: WPRE forward primer: 5′-TGCCCGCTGCTGGAC-3′; WPRE reverse primer: 5′-CCGACAACACCACGGAATTG-3′; and WPRE probe: 5′-CAGTGCCCAACAGCC-3′ (1.0–6.0 × 10^12^ viral genomes (vg)/ml, TaqMan Assay, Applied Biosystems). In addition, the filling rate of the rAAV preparation was determined by electron microscopy. Purified virus samples were applied to carbon-coated electron microscopy grids, negatively stained with 1% uranyl acetate and rinsed with ultrapure water. Representative images of selected areas were taken using transmission electron microscopy at 6,3000x magnification (Zeiss 912 Omega Transmission Electron Microscope). The filling rate was determined by manually counting filled and empty particles using ImageJ (NIH). Empty capsids were distinguished from filled capsids, resulting from the uptake of uranyl acetate in the capsid core or from collapse staining of the particle. The genomic titers (genome copies (gc)/ml) and the filling rate (%) were as follows: rAAV-BMP7-2A-Venus, 1.69 × 10^12^, 88%; rAAV-BMP2-2A-Venus, 1.86 × 10^12^, 92%; rAAV-Noggin-2A-VENUS, 1.02 × 10^12^, 49%; rAAV-Venus, 2.90 × 10^12^, 64%; and for rAAV-sVenus, 5.96 × 10^12^, 98%. The infectivity was determined in primary neurons. Primary cortical neurons were infected with rAAVs on DIV 4 at multiplicities of infection (MOI) of 2 × 10^3^ gc/cell. The expression of rAAV-transduced proteins in rat primary neurons was evaluated on DPI 7 by Venus fluorescence, immunoblots, immunoassay, or immunostainings [27,44].

### BMP stimulation of HeLa cells

HeLa cells were incubated at 37°C and 5% CO_2_ in a humidified atmosphere. For stimulation experiments HeLa cells were plated in 12-well plates in MEM containing 5% FCS (Invitrogen) at a density of 1.5 × 10^5^ in 1 ml/well. The following day medium was changed to serum-free MEM. On the third day medium was replaced by 1 ml conditioned medium taken from infected primary cortical neurons or 1 ml DMEM containing 100 ng/μl recombinant proteins (r-BMP7, r-BMP2 and r-Noggin, R&D Systems) to stimulate HeLa cells.

### Primary cortical neuron culture

Cultures of primary cortical neurons were prepared from cerebral cortices of E16 NMRI mice as described previously [45]. Briefly, cortices were dissected in ice-cold PBS supplemented with Hepes (7.38 g/l) and glucose (6 g/l). Pooled cortical tissues were dissociated by incubation with trypsin-EDTA for 8 min at 37°C, followed by mechanical trituration using a fire-polished Pasteur pipette. After resuspension in Neurobasal (NB) medium containing 1 × B27, 0.5 mM L-glutamine, 100 U/ml penicillin, and 100 μg/ml streptomycin, 2 × 10^5^ cells per well were plated in 24-well plates precoated with poly-D-lysine (50 μg/ml). On DIV 1 half of the medium was exchanged by fresh NB medium containing 5% fetal bovine serum. Then, on DIV 4 and DIV 8 half of the medium was exchanged with fresh NB until DIV 10–12 when cells were used for experiments. On DIV 11 half of the medium was exchanged with fresh medium containing r-BMP7 (R&D Systems). Thirty minutes later, H_2_O_2_ was added to the medium for 24 hours. Culture media were collected to measure levels of lactate dehydrogenase (LDH) using the cytotoxicity detection kit (Roche). Here, six wells were analyzed per condition and the assays were performed in duplicate. Per well, 75 μl of supernatant was added to 75 μl of assay reagent and incubated for 30 min at room temperature. LDH activity was determined by colorimetry according to the LDH assay kit’s instructions and expressed as percentage of LDH activity of untreated cells (LDH %Control).

### Constitutive release of v- BMP7, v-BMP2 and v-Noggin in PCN

Primary cortical neurons (PCN) were cultured as described above. On DIV 4 PCN were infected with rAAV-BMP7-2A-Venus, rAAV-BMP2-2A-Venus or rAAV-Noggin-2A-Venus at a MOI of 1 × 10^3^ gc/cell. Lysates (L, 4 μl) and supernatants (S, 12 μl) were collected and analyzed in immunoblots or by ELISA.

### Characterization of rAAV in immunoblots

Infected primary cortical neurons were harvested with RIPA lysis buffer (50 mM Tris–HCl, pH 7.6; 150 mM NaCl; 1% NP-40; 0.5% sodium deoxycholate; 0.1% SDS) containing protease inhibitor cocktail (Complete, Roche) 7 days after rAAV infection. Brain tissue (cortex and hippocampus) was collected 8 weeks after virus injection and homogenized in 25 mM Hepes buffer containing a protease inhibitor cocktail on ice. Proteins were separated by SDS-PAGE (10% separating and 4% stacking gels) and transferred onto nitrocellulose membranes. Immunoblots were probed with monoclonal mouse anti-GFP (1:8000, Clontech), polyclonal rabbit anti-2A (1:2000, Millipore), polyclonal rabbit anti-phospho-Smad1/5/8 (1:1000, Cell Signaling Technology), polyclonal rabbit anti-BMP7 (1:1000, Abcam), and monoclonal mouse anti-β-actin (1:5000) antibodies. Horseradish peroxidase-linked anti-rabbit or anti-mouse was used as secondary antibodies (1:15,000, Vector Laboratories), and blots were visualized by enhanced chemiluminescence (ECL kit, GE Healthcare). Immunoblots were quantified using ImageJ (NIH).

### Characterization of rAAV expression by immunostaining

Cultured primary cortical neurons on DPI 7 or brains from virus-injected mice on DPI 56 were fixed with 4% paraformaldehyde (PFA), and 100-μm sagittal sections were obtained from brains. Immunostaining was performed with polyclonal rabbit anti-2A (1:1000) or polyclonal chicken anti-GFP (1:5000, Abcam) and Cy3-coupled anti-rabbit or FITC-coupled anti-chicken secondary antibodies (1:400, Jackson ImmunoResearch).

### Stereotaxic rAAV delivery into brains of adult mice

Stereotaxic rAAV injection into adult C57BL6/N mice was performed according to a protocol described previously [46]. The following coordinates relative to bregma were used for injection: anteroposterior −2 mm; mediolateral ±1.5 mm; dorsoventral from pial surface −1.55 mm for DG, −1.05 mm for CA1, −0.35 for CTX. In brief, animals were anesthetized with an intraperitoneal injection of a mixture of ketamine hydrochloride (Inresa Arzneimittel; 100 mg per kilogram body weight) and xylazine (Inresa Arzneimittel; 10 mg per kilogram body weight) in PBS. After loss of reflexes the animal was fixed in a small animal stereotaxic frame (David Kopf instruments) and eyes were covered with bepanthene. The skull was exposed and levels were adjusted using the Kopf digital display console (David Kopf instruments). Coordinates were set and a small craniotomy was made at the defined position into the skull using an OS-40 drilling device (Osada Electronic). Virus solution was sucked into a glass micropipette with an inner diameter of 9 μm by applying back-pressure and a total volume of 264 nl was injected into the three different injection sites at a rate of 50 nl/min. After injection, the micropipette was kept in place for 5 min to avoid backflow of the injected virus during micropipette retraction. The scalp was sutured, and the animal was placed on a heating pad until recovery from surgery and then returned to its home cage.

### BRE: GFP mice

Adult BRE: GFP reporter mice [35] were analyzed on DPI 10 after rAAV or PBS injection in the cortex and hippocampus. Coronal sections were immunostained as described above using mouse anti-GFP (1:1000, Clontech) and Cy3-anti-mouse (Jackson Immunoresearch) for enhanced GFP detection. The Cy3 fluorescence was converted to the green channel in the RGB color mode in Photoshop.

### Stereotaxic rAAV delivery into brains of newborn mice

Stereotaxic rAAV injection into neonate C57BL6/N mice at P0 was performed as described previously [37]. The following coordinates relative to lambda were used for injection: Lateral ventricle (LV), rostral 1 mm, lateral 1 mm and ventral 1.5 mm; HPC, rostral 2 mm, lateral 0.7 mm and ventral 1.5 mm. Briefly, newborn pubs were separated from their mother and cryoanesthetized for 5 min. Purified rAAV1/2 were infused into LV and HPC (1 μl virus per injection site) of neonates according to the defined coordinates at 200 nl/sec through a 33-gauge beveled needle via microprocessor-controlled WPI (World Precision Instruments) infusion-pump driving a 100 μl syringe. After injection, the hand-held needle was kept in place for additional 3 s to avoid backflow of the injected virus. Following injection the pups were collected on a 37°C warm plate for recovery. After regaining normal body color and full mobility, the injected pups were rolled in home cage bedding and returned to their mother as a group. Injected animals were kept until experiments were performed.

### Middle cerebral artery occlusion (MCAO)

Eight-week-old male C57BL/6 N mice that had been infected with rAAV vectors on P0 were anesthetized by intraperitoneally injecting 15 μl of 2.5% avertin per g body weight. Middle cerebral artery occlusion (MCAO) was performed as described previously [47,48]. A skin incision was made between the ear and the orbit on the left side and the temporal muscle was removed by electrical coagulation. Then, the MCA was exposed and occluded by microbipolar coagulation. Rectal temperature was maintained at 37°C during surgery by a heating pad and monitored continuously. After 48 hours of MCAO, mice were deeply anesthetized with avertin and perfused transcardially with Ringer’s solution. Brains were removed and immediately frozen on dry ice. Serial coronal cryosections (20 μm in thickness) were cut every 400 μm and stained with a silver technique [48]. Stained sections were scanned at 300 d.p.i., and the infarct area was measured using ImageJ (NIH). To correct for edema formation, the difference between areas of the left and right hemispheres was subtracted from the measured silver-negative area [49]. For calculating the infarct volume, the infarct areas were added and multiplied by the distance between the sections (400 μm). Based on previous MCAO experiments in our laboratory we calculated that, in experiments with four groups, 12 mice per group were required to detect a 40% change of infarct size with a power of >0.8 and a p < 0.05 by one-way ANOVA. Experimenters were blinded for the treatment group.

### TUNEL staining and quantification

For quantification of apoptosis after MCAO, terminal deoxynucleotidyl transferase- mediated dUTP-biotin nick end labeling (TUNEL) was performed on cryosections using the in-situ-cell death detection kit (Roche). Sections were air-dried for 10 min at RT and postfixed in 4% PFA for 30 min. Then, sections were washed twice in PBS for 5 min and subsequently permeabilized in 0.1% sodium citrate, 0.1% TrtionX-100 in PBS. Sections were washed twice in PBS for 5 min and incubated with TUNEL reaction mix (50 μl per section, TUNEL-enzyme: labeling solution, 1:7) in a humid chamber for 2 h in the dark. After washing in PBS and DAPI-staining for 5 min at RT sections were washed in PBS and mounted with Mowiol/DABCO (Merck/Sigma-Aldrich). For combined TUNEL and fluorescence immunostaining, immunohistochemistry was performed as described above. To quantify the percentage of apoptotic cells fluorescence images were acquired at 10x magnification in three optical fields of the penumbra and analyzed using ImageJ (NIH). The number of DAPI and TUNEL positive cells was counted manually. Experimenters were blinded for the treatment group.

### ELISA assays

Culture supernatants or lysates of infected PCN were collected and BMP2-immunoassay was performed in a 96-well plate according the manufacturer’s instructions (R&D systems). The optical density of each well was measured at 450 nm using the VersaMax microplate reader (Molecular Devices). Protein concentrations were determined according to appropriate BMP2 standard curves.

### Statistical analysis

Data were analyzed using GraphPad Prism 5 (Graphpad) and results are presented as mean ± standard error of the mean (SEM) of the indicated number of independent replicates unless otherwise indicated. Two-tailed Student’s t test was used for comparisons between two independent groups. For comparing more than two groups one way analysis of variance (ANOVA) was performed followed by Turkey’s multiple comparison post hoc test.

## Abbreviations

ANOVA: Analysis of variance; bGHpA: Bovine growth hormone polyadenylation; BMPs: Bone morphogenic proteins; CAG: Chicken beta-actin enhanced cytomegalovirus promoter; CNS: Central nervous system; CTX: Cortex; DAPI: 4′; 6-diamidino-2-phenylindole; DIV: Days *in vitro*; DPI: Day post infection; DG: Dentate gyrus; DV: Dorsoventral; dUTP: Deoxyuridine triphosphate; EDTA: Ethylenediaminetetraacetic acid; ELISA: Enzyme linked immunosorbent assay; FITC: Fluorescein isothiocyanate; GFP: Green fluorescent protein; HPC: Hippocampus; ITRs: Inverted terminal repeats; LDH: Lactate dehydrogenase; LV: Left ventricle; MCAO: Middle cerebral artery occlusion; MCA: Middle cerebral artery; MOI: Multiplicities of infection; MW: Molecular weight; NB: Neurobasal; OB: Olfactory bulb; PBS: Phosphate buffered saline; PCN: Primary cortical neurons; PFA: Paraformaldehyde; PN: Primary neurons; rAAV: Recombinant adeno-associated virus; rAAVs: Recombinant adeno-associated viruses; r-BMP: Recombinant-bone morphogenic protein; RT: Room temperature; SDS-PAGE: Sodium dodecyl sulfate polyacrylamide gel electrophoresis; SEM: Standard error of the mean; TGFβ: Transforming growth factor beta; TUNEL: Terminal deoxynucleotidyl transferase dUTP nick end labeling; v-BMP: Viral expressed-bone morphogenic protein; WPRE: Woodchuck posttranscriptional regulatory element

## Competing interests

The authors declare that they have no competing interests.

## Authors’ contributions

AH participated in the design and coordination of the study, generated and injected the viruses, performed the *in-vitro* work, assisted in the surgery on mice and drafted the manuscript. MR performed surgery on mice and measured cerebral blood flow. GD helped to draft the manuscript. SW participated in the design of the study. HJ performed the BRE: GFP experiments. RS conceived of the study, participated in its design and wrote the manuscript. MS participated in the design, coordination of the study, and wrote the manuscript. All authors read and approved the final manuscript.
